# Supplementary material for: Delayed entanglement echo for individual control of a large number of nuclear spins
Source: Nat Commun. 2017 Mar 3;8:14660. doi: 10.1038/ncomms14660 (PMC5338027; doi:10.1038/ncomms14660)
Supplement: Supplementary Information — Supplementary Figures, Supplementary Notes and Supplementary References. [file ncomms14660-s1.pdf]

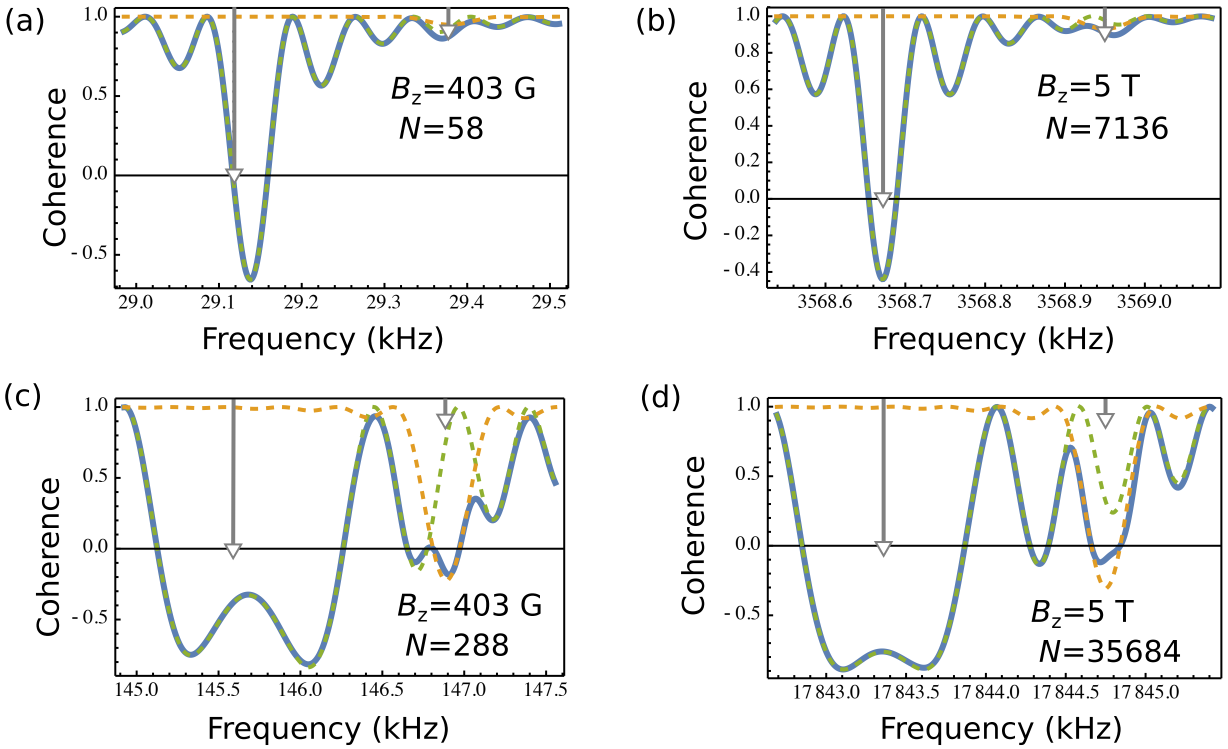

Supplementary Figure 1. **Coherence signals obtained by CP sequences.** Coherence of NV electron spin (blue solid line) as a function of DD frequency $\omega_{\rm DD} = \pi/\tau_{\rm CP}$ (with $\tau_{\rm CP}$ the pulse intervals of CP sequences), under the control of N-pulse CP sequences at a magnetic field $B_{\rm z}$. The CP sequences have durations of $\sim$ 1 ms using the pulse numbers and magnetic fields indicated on the figures. The green and yellow dashed lines are the signals of single spins. The arrows indicate the frequencies $\omega_j/k_{\rm DD}$ with the lengths proportional to $A_j^{\perp}$. The signals of resonances at $k_{\rm DD} = 15$ in (a) and (b) are dominated by the nuclear spin with a strong $A_j^{\perp}$. While in (c) and (d), increasing the interactions by using a smaller $k_{\rm DD} = 3$ also increases the interference from the spin with a strong $A_j^{\perp}$, prohibiting the spin addressing on the nuclear spin with a weaker $A_j^{\perp}$. In (b) and (d), the number of pulses increases under a stronger magnetic field, compared with the cases in (a) and (c).

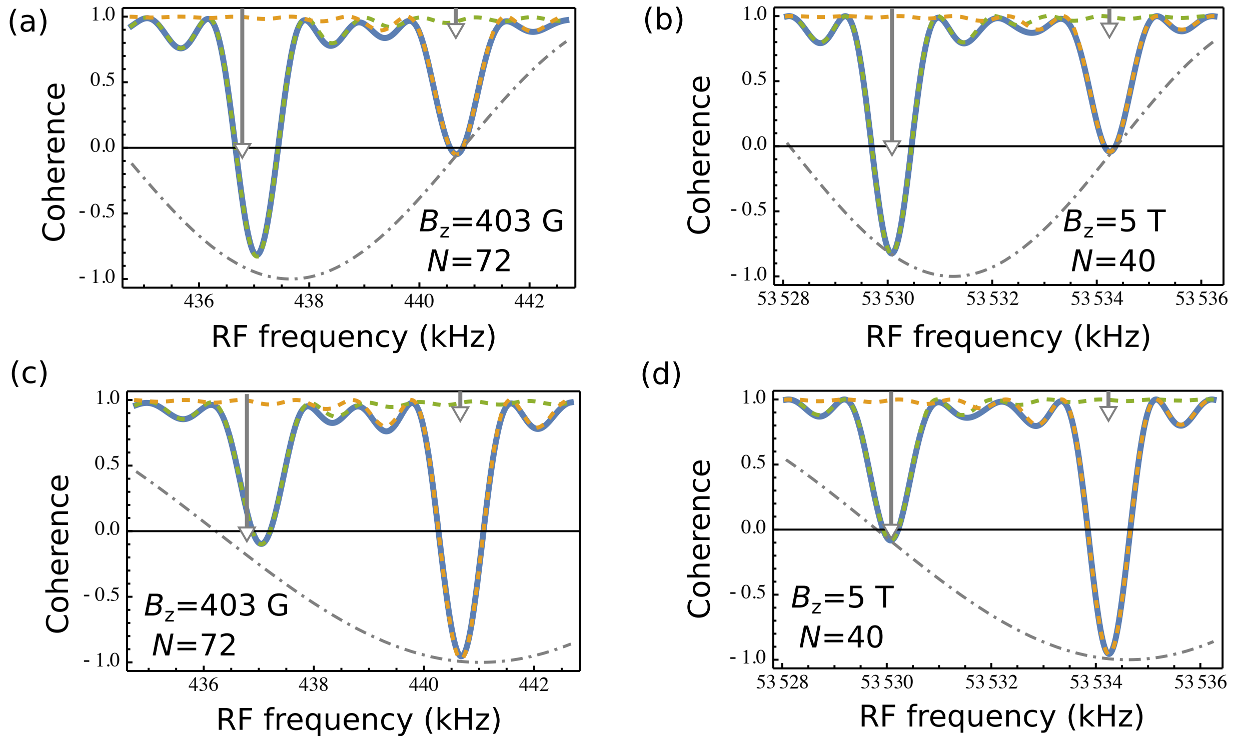

Supplementary Figure 2. **Coherence signals obtained by delayed entanglement echoes.** Coherence of NV electron spin (blue solid line) as a function of the frequency $\omega_{\rm rf}$ of RF driving at the delay window, which the duration $t_{\rm rf} \approx 1$ ms and $\theta_{\rm rf} = \pi$. The pulse number N of CP sequences used to protect the delay window are indicated on the figures, as well as the magnetic filed B_z. The green and yellow dashed lines are the signals of single spins. Each of the arrow located at the nuclear precession frequency ω_j has a length proportional to $A_j^{\perp}$. The interaction times $\tau = 20$ μs for (a) and (b), while a shorter $\tau = 13$ μs for (c) and (d). Dash-dotted lines are the curve $\cos[4\tau(\omega_{\rm rf} - \omega_{^{13}{\rm C}})]$. The qubit levels $m_{\rm s} = \pm 1$ are used at the interaction windows.

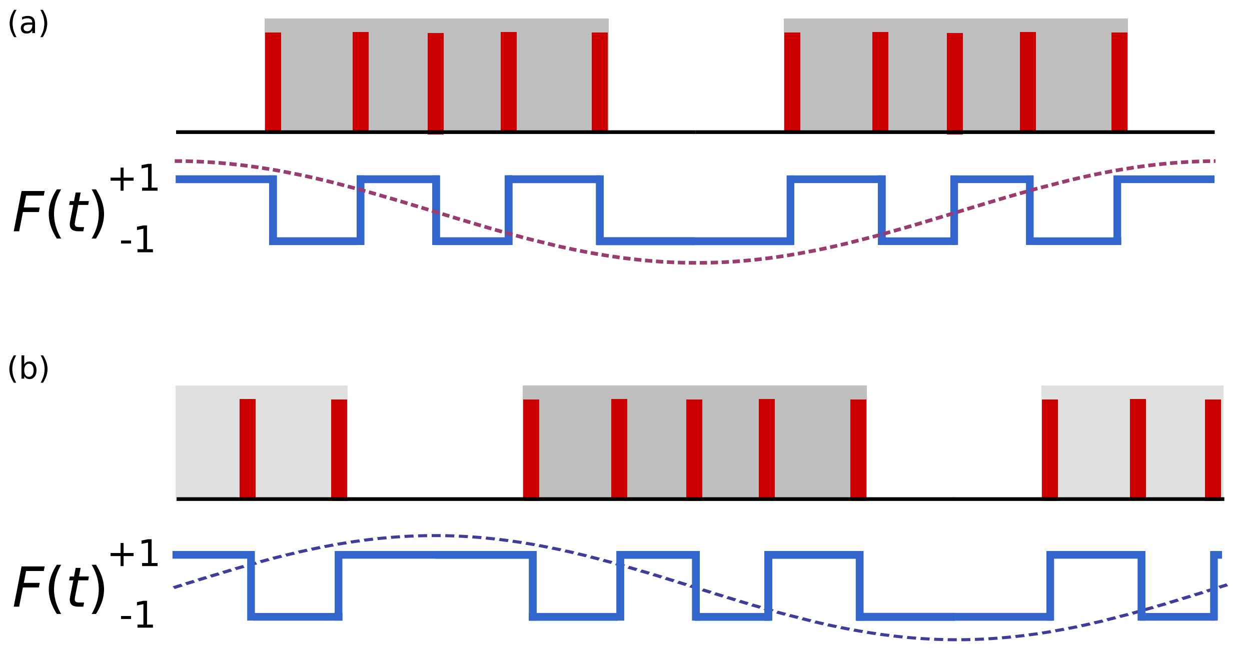

Supplementary Figure 3. **Illustration of AXY pulse sequences in one period.** (a) Symmetric version and its corresponding modulation function shown below. (b) The anti-symmetric counterpart. Each of the shaded areas with a heavier colour highlights a composite π pulse in the AXY sequences. The dashed lines indicate the sinusoidal signals on-resonant to the modulation function $F(t)$.

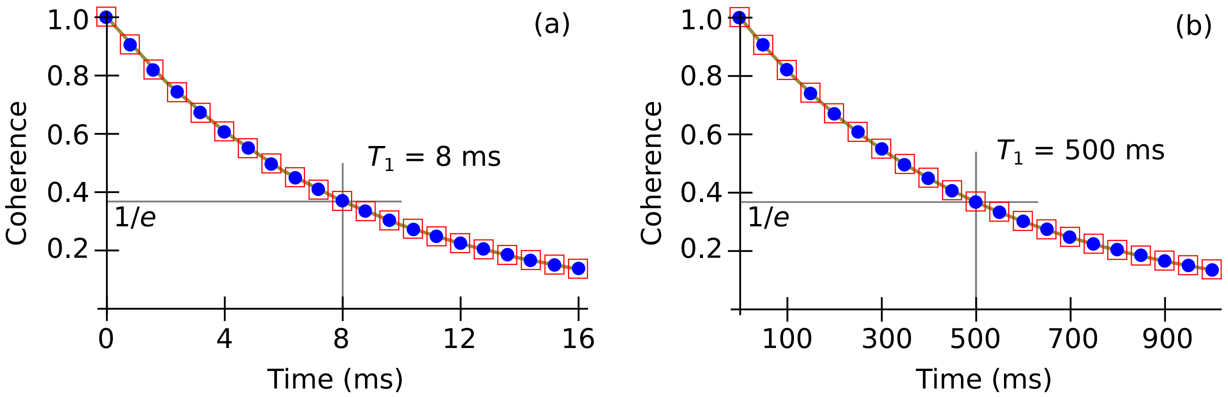

Supplementary Figure 4. **Coherence time of nuclear memories.** Squares (dots) are the coherence of nuclear memory ^{14}N (^{13}C) qubit simulated by a Lindblad master equation with the NV electron spin initialized in the state $|+1\rangle$ for a room-temperature electron relaxation time $T_1 = 8$ ms (a) and for a low-temperature $T_1 = 500$ ms (b). The ^{13}C spin memory has the hyperfine components $A_{\rm m}^{\parallel} = -2\pi \times 31.26$ kHz and $A_{\rm m}^{\perp} = 2\pi \times 29.24$ kHz. The solid lines are the results of a qubit under a classical fluctuating magnetic field.

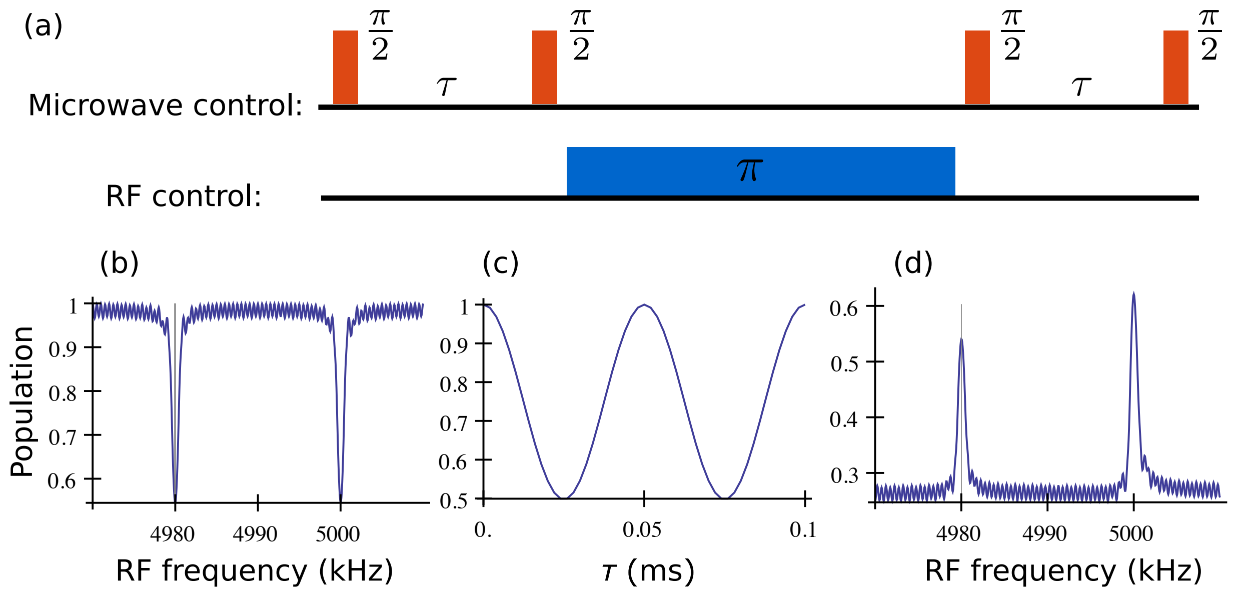

Supplementary Figure 5. **Population signal of Mims ENDOR.** (a) The protocol of Mims ENDOR. The microwave control shown in the upper panel is the stimulated echo sequence with non-selective microwave $\pi/2$ pulses. (b) The signal of Mims ENDOR when there is only one ^{13}C nuclear spin, with the hyperfine field $A_1^{\parallel} = 2\pi \times 19.98$ kHz and $A_1^{\perp} = 2\pi \times 7.66$ kHz. The length of RF pulse is 1 ms. (c) The same as (b) but plotted as a function of the time τ under an on-resonant RF π pulse with the frequency denoted by the vertical line in (b). (d) The signal under the same control parameters as (b), when there is an additional ^{13}C nuclear spin with distinct hyperfine field $A_2^{\parallel} = 2\pi \times 42.8$ kHz and $A_2^{\perp} = 2\pi \times 23$ kHz. Although the intrinsic ^{14}N is fully polarized to $m_{\mathrm{N}} = -1$, the Mims ENDOR still cannot address nuclear spins because unwanted spin interactions are not compensated.

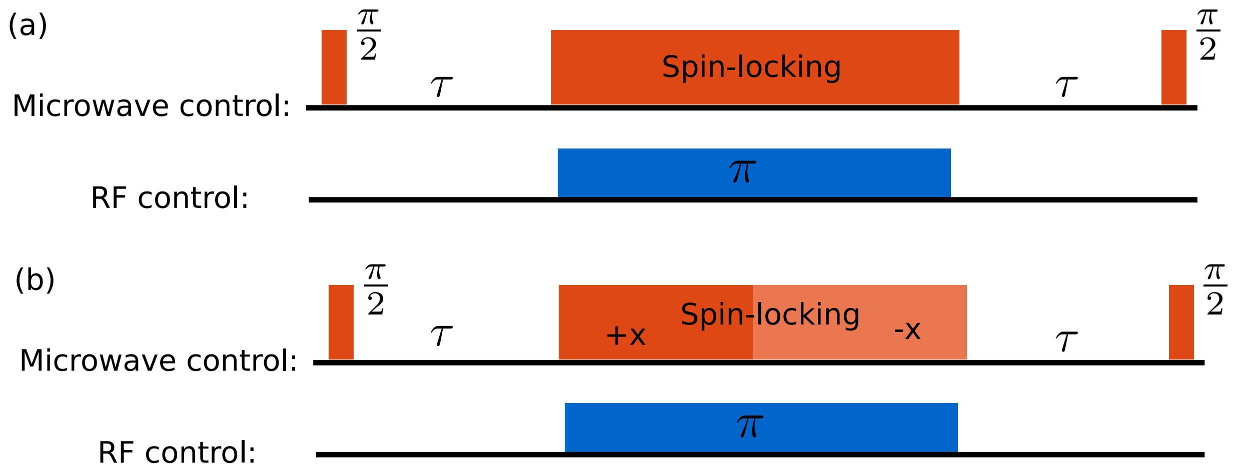

Supplementary Figure 6. **Hyperfine-decoupled ENDOR.** (a) Hyperfine-decoupled ENDOR. (b) PEANUT ENDOR.

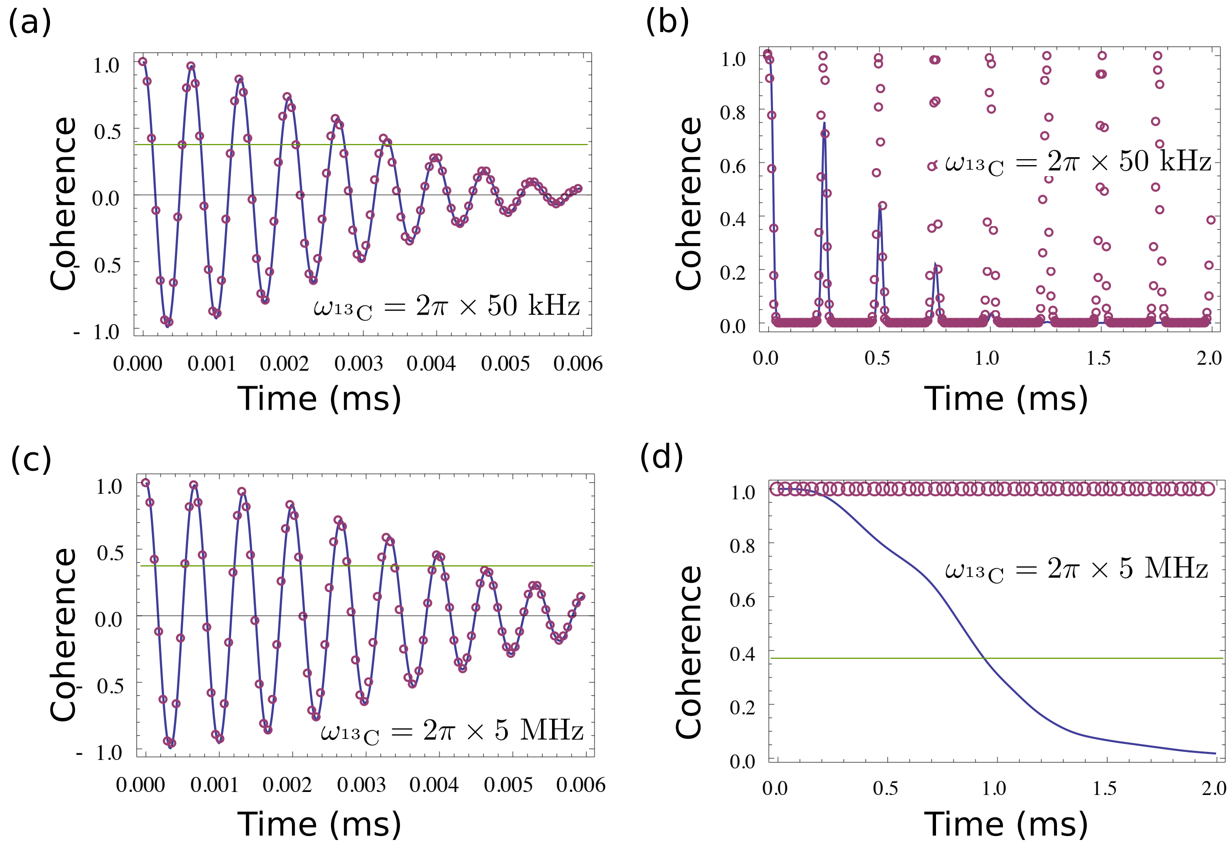

Supplementary Figure 7. **Coherence of NV electron spin in interacting and non-interacting spin baths.** (a) Free evolution of the coherence (blue solid line) of NV electron spin in a spin bath used in Fig. 2 of the main text. (b) Coherence of NV electron spin with the application of a spin echo pulse at the middle of the evolution time (blue solid line). (c) As in (a), but with a much stronger magnetic field. (d) As in (b), but with a magnetic field as in (c). In (a)-(d), the circles corresponds to the case without nuclear-nuclear interactions. Under strong magnetic fields and without nuclear-nuclear interactions, the NV electron spin can have a much longer coherence time.

Supplementary Note 1: Hamiltonian of NV centre and nuclear spins

Under a magnetic field $\mathbf{B} = B_z\hat{\mathbf{z}}$ along the NV symmetry axis, the Hamiltonian of NV centre electron spin and its nuclear environment reads ($\hbar = 1$)

$$H = H_{\rm NV} + H_{\rm nZ} + H_{\rm hf} + H_{\rm nn}. \tag{1}$$

Here $H_{\rm NV} = DS_z^2 - \gamma_e B_z S_z$ is the electron spin Hamiltonian with the spin operator $S_z = \sum_{m_s=\pm1,0} m_s|m_s\rangle\langle m_s|$, the ground state zero field splitting $D \approx 2\pi \times 2.87$ GHz, and $\gamma_e = -2\pi \times 2.8$ MHz G^{-1} the electron spin gyromagnetic ratio[1]. The nuclear Zeeman Hamiltonian $H_{\rm nZ} = -\sum_j \gamma_j \mathbf{B}\cdot\mathbf{I}_j$, where γ_j is the nuclear gyromagnetic ratio and $\mathbf{I}_j$ is the spin operator for the j-th nuclear spin. The dipole-dipole interactions between nuclear spins are

$$H_{\rm nn} = \sum_{j>k} \frac{\mu_0}{4\pi}\frac{\gamma_j\gamma_k}{|\mathbf{r}_{j,k}|^3}\left[\mathbf{I}_j\cdot\mathbf{I}_k - \frac{3(\mathbf{I}_j\cdot\mathbf{r}_{j,k})(\mathbf{r}_{j,k}\cdot\mathbf{I}_k)}{|\mathbf{r}_{j,k}|^2}\right], \tag{2}$$

with μ_0 being the vacuum permeability, $\mathbf{r}_{j,k} = \mathbf{r}_j - \mathbf{r}_k$ the difference between the k-th and j-th nuclear positions. Typically the electron-nuclear flip-flop terms in the hyperfine interaction $H_{\rm hf}$ are suppressed by the large energy mismatch between electron and nuclear spins, giving $H_{\rm hf} = S_z\sum_j \mathbf{A}_j\cdot\mathbf{I}_j$ under the secular approximation. However, for strong hyperfine interactions the virtual flips of the electron spin could cause observable effects, an aspect that we will discuss later (see Supplementary Note 3). For nuclear spins not too close to the NV centre, the hyperfine interaction takes the dipolar form and the hyperfine field

$$\mathbf{A}_j = \frac{\mu_0}{4\pi}\frac{\gamma_e\gamma_j}{|\mathbf{r}_j|^3}\left(\hat{\mathbf{z}} - \frac{3\hat{\mathbf{z}}\cdot\mathbf{r}_j\mathbf{r}_j}{|\mathbf{r}_j|^2}\right). \tag{3}$$

The total Hamiltonian under secular approximation is diagonal with respect to the electron spin states $|m_s\rangle$.

We choose two of the three states as the qubit basis states for the NV electron spin. When we use the manifold of the electron spin states $|\uparrow_e\rangle = |+1\rangle$ and $|\downarrow_e\rangle = |-1\rangle$, we have the Hamiltonian

$$H = (D - \gamma_e B_z)\,\sigma_z + \sigma_z\sum_j \mathbf{A}_j\cdot\mathbf{I}_j - \sum_j \gamma_j\mathbf{B}\cdot\mathbf{I}_j + H_{\rm nn}. \tag{4}$$

where the Pauli operator $\sigma_z = |\uparrow_e\rangle\langle\uparrow_e| - |\downarrow_e\rangle\langle\downarrow_e|$. In the rotating frame of $(D - \gamma_e B_z)\,\sigma_z$, the Hamiltonian Eq. (4) becomes

$$H_1 = \sigma_z\sum_j \mathbf{A}_j\cdot\mathbf{I}_j - \sum_j \gamma_j\mathbf{B}\cdot\mathbf{I}_j + H_{\rm nn}. \tag{5}$$

Note that the nuclear Hamiltonian is not shifted by the hyperfine fields, because the average of m_s for the used electron-spin levels vanishes and in the limit of fast electron spin flipping the averaged magnetic field is the external one $\mathbf{B}$.

On the other hand, when we work in the manifold of the electron spin states $|\uparrow_e\rangle = |+1\rangle$ and $|\downarrow_e\rangle = |0\rangle$, we have the Hamiltonian

$$H_{\frac{1}{2}} = \frac{1}{2}\sigma_z\sum_j \mathbf{A}_j\cdot\mathbf{I}_j - \sum_j\left(\gamma_j\mathbf{B} - \frac{1}{2}\mathbf{A}_j\right)\cdot\mathbf{I}_j + H_{\rm nn}, \tag{6}$$

in the rotating frame of $(D - \gamma_e B_z)\,|\uparrow_e\rangle\langle\uparrow_e|$.

In summary, we write the Hamiltonian for both qubit-manifolds as

$$H_\eta = \eta\sigma_z\sum_j \mathbf{A}_j\cdot\mathbf{I}_j + H_{{\rm n},\eta} + H_{\rm nn}. \tag{7}$$

The coupling constant $\eta \equiv (\max m_s - \min m_s)/2 = 1/2$ for the manifold of the electron spin levels $m_s = 0, +1$, while $\eta = 1$ for the levels $m_s = \pm1$. The nuclear Hamiltonian describing nuclear precession reads

$$H_{{\rm n},\eta} = -\sum_j(\gamma_j\mathbf{B} - c_\eta\mathbf{A}_j)\cdot\mathbf{I}_j \equiv -\omega_j\hat{\boldsymbol{\omega}}_j\cdot\mathbf{I}_j, \tag{8}$$

where the unit vectors $\hat{\boldsymbol{\omega}}_j$ denote the directions of $\gamma_j\mathbf{B} - c_\eta\mathbf{A}_j$. Here c_η is the average of the quantum numbers m_s of the electron spin levels used in the qubit manifold. That is, $c_\eta = 0$ for the electron qubit manifold $m_s = \pm1$, while

$c_\eta = 1/2$ for the manifold $m_{\rm s} = 0, +1$. For the Hamiltonian H_1 ($m_{\rm s} = \pm 1$ and $\eta = 1$), the electron-nuclear coupling is stronger and the nuclear precession frequencies $\omega_j = \gamma_j B_{\rm z}$ are the bare nuclear Larmor frequencies. While for the Hamiltonian $H_{\frac{1}{2}}$ ($m_{\rm s} = 0, +1$ and $\eta = 1/2$), the electron-nuclear coupling is weaker and the precession frequencies $\omega_j = |\gamma_j \mathbf{B} - \frac{1}{2}\mathbf{A}_j|$ are shifted by the hyperfine field at the positions of the nuclear spins. When the electron spin is decoupled from the nuclear spins (e.g., by dynamical decoupling as will be discussed in the next section) and the nuclear-nuclear coupling can be neglected, the nuclear spins precess freely with their frequencies ω_j.

In the rotating frame of nuclear spin precession $H_{{\rm n},\eta}$, the interaction Hamiltonian $\eta\sigma_{\rm z}\sum_j \mathbf{A}_j \cdot \mathbf{I}_j$ becomes[2]

$$H_{\rm int} = \eta\sigma_{\rm z}\sum_j \left[\mathbf{A}_j^{\rm x}\cos(\omega_j t) + \mathbf{A}_j^{\rm y}\sin(\omega_j t) + \mathbf{A}_j^{\rm z}\right]\cdot \mathbf{I}_j, \tag{9}$$

with

$$\mathbf{A}_j^{\rm x} \equiv \mathbf{A}_j - \mathbf{A}_j^{\rm z}, \tag{10}$$
$$\mathbf{A}_j^{\rm y} \equiv \hat{\boldsymbol{\omega}}_j \times \mathbf{A}_j, \tag{11}$$
$$\mathbf{A}_j^{\rm z} \equiv \mathbf{A}_j \cdot \hat{\boldsymbol{\omega}}_j \hat{\boldsymbol{\omega}}_j. \tag{12}$$

The hyperfine components have the strengths $|\mathbf{A}_j^{\rm x}| = |\mathbf{A}_j^{\rm y}| = A_j^{\perp}$ and $|\mathbf{A}_j^{\rm z}| = A_j^{\parallel}$. The time-dependent terms in Eq. (9) do not commute with the nuclear precession $H_{{\rm n},\eta}$.

Under a strong magnetic field $B_{\rm z} \gg A_j^{\perp}$, $\hat{\boldsymbol{\omega}}_j \approx \hat{\mathbf{z}}$. The nuclear spin flips are suppressed, giving

$$H_{\rm int} \approx \eta\sigma_{\rm z}\sum_j \mathbf{A}_j^{\rm z}\cdot\mathbf{I}_j = \eta\sigma_{\rm z}\sum_j A_j^{\parallel} I_j^{\rm z}. \tag{13}$$

If we apply Lee-Goldburg (LG) off-resonance control[3], we can achieve similar Hamiltonians[4,5]

$$H_{\rm int}^{\rm LG} \approx \eta\sigma_{\rm z}\sum_j A_j^{\parallel}\cos\gamma_j \tilde{I}_j^{\rm z}, \tag{14}$$

where $\tilde{I}_j^{\rm z} = \hat{\boldsymbol{\nu}}_j \cdot \mathbf{I}_j$ with $\hat{\boldsymbol{\nu}}_j$ the unit vector denoting the nuclear precession in the frame of LG control. The projection factor $\cos\gamma_j = \hat{\boldsymbol{\omega}}_j \cdot \hat{\boldsymbol{\nu}}_j \approx 1/\sqrt{3}$.

The interaction Hamiltonian Eq. (13) commutes with the nuclear precession. Similarly, Eq. (14) commutes with the nuclear precession around $\hat{\boldsymbol{\nu}}_j$ in the frame of LG control. Combined with the delay entanglement control described in the main text, we can keep only terms on the target spins in $H_{\rm int}$ or $H_{\rm int}^{\rm LG}$. The effective electron-nuclear interactions by delay entanglement control do not broaden the nuclear precession frequencies for addressing.

Supplementary Note 2: Remarks on spin addressing by dynamical decoupling

a. *Effective interaction Hamiltonians under dynamical decoupling*

Nuclear spins can be addressed by dynamical decoupling (DD)[2,4,6–10]. The DD pulses flip the NV electron qubit. After application of n π pulses, $\sigma_{\rm z} \to F(t)\sigma_{\rm z}$ with the modulation function $F(t) = (-1)^n$. We consider periodic sequences with $F(t) = F(t + 2\pi/\omega_{\rm DD})$ in this work. The interaction Hamiltonian Eq. (9) becomes

$$H_{\rm int} = \eta F(t)\sigma_{\rm z}\sum_j \left[\mathbf{A}_j^{\rm x}\cos(\omega_j t) + \mathbf{A}_j^{\rm y}\sin(\omega_j t) + \mathbf{A}_j^{\rm z}\right]\cdot \mathbf{I}_j. \tag{15}$$

This instantaneous-pulse control changes the electron-nuclear dynamics[7,11,12]. To get insight on nuclear spin sensing by DD pulse sequences, we expand the modulation function in a Fourier series,

$$F(t) = \sum_{k\geq 1}[f_k^{\rm s}\cos(k\omega_{\rm DD}t) + f_k^{\rm a}\sin(k\omega_{\rm DD}t)],$$

using that DD pulses have been designed to remove static noise and hence there is no static term in the Fourier series. For periodic symmetric sequences $f_k^{\rm a} = 0$. The frequency $\omega_{\rm DD}$ characterises the flipping rate of the NV electron qubit. For example, for the traditional Carr-Purcell (CP) sequence[13] and its variations[14–16] having a time interval

τ_{CP} between successive π pulses, $\omega_{\mathrm{DD}} = \pi/\tau_{\mathrm{CP}}$, $f_k^{\mathrm{s}} = 4(k\pi)^{-1}\sin(k\pi/2)$, and $f_k^{\mathrm{a}} = 0$. The expansion coefficients can be tuned by adaptive XY (AXY) sequences[2].

A nuclear spin with the precession frequency ω_n can be addressed by resonance to the k_{DD} harmonic of the driving rate, that is, $\omega_n = k_{\mathrm{DD}}\omega_{\mathrm{DD}}$. With the additional conditions (with $j \neq n$) $|\gamma_j B_{\mathrm{z}}| \gg k_{\mathrm{DD}}|\mathbf{A}_j|$ and

$$|\omega_n - \omega_j| \gg |f_{k_{\mathrm{DD}}} A_j^{\perp}|, \tag{16}$$

we have single spin addressing under periodic symmetric sequences $H_{\mathrm{int}} \approx (\eta/2) f_{k_{\mathrm{DD}}}^{\mathrm{s}} A_j^{\perp}\sigma_{\mathrm{z}} I_j^{\mathrm{x}}$[2]. Similar addressing Hamiltonians ($H_{\mathrm{int}}^{\mathrm{LG}} \propto \sigma_{\mathrm{z}}\tilde{I}_j^{\mathrm{x}}$ with $\tilde{I}_j^{\mathrm{x}}$ a spin operator projected perpendicular to $\hat{\boldsymbol{\nu}}_j$) can be achieved under LG control[4,17].

Nuclear spins can also be addressed by continuous DD[9]. In the rotating frame of a constant microwave driving $\Omega_{\mathrm{e}}\sigma_{\mathrm{x}}/2$ with the Rabi frequency Ω_{e} (the frequency of nuclear spin precession in the spin-lock frame), the Pauli operator of NV electron qubit transforms as $\sigma_{\mathrm{z}} \to \sigma_{\mathrm{z}}\cos(\Omega_{\mathrm{e}}t) + \sigma_{\mathrm{y}}\sin(\Omega_{\mathrm{e}}t)$. The driving rate of the electron spin is $\omega_{\mathrm{DD}} = \Omega_{\mathrm{e}}$. When Ω_{e} is on-resonance to the nuclear spin precession frequency ω_j, that is, $\Omega_{\mathrm{e}} = \omega_j$, we have the addressing Hamiltonian $H_{\mathrm{int}} \approx (\eta/2)A_j^{\perp}(\sigma_{\mathrm{z}} I_j^{\mathrm{x}} + \sigma_{\mathrm{y}} I_j^{\mathrm{y}})$ when $|\gamma_j B_{\mathrm{z}}| \gg |\mathbf{A}_j|$ and $|\omega_n - \omega_j| \gg A_j^{\perp}$ for $j \neq n$.

b. Dynamics under the control of Carr-Purcell sequence

We analyse the system dynamics from another picture of conditional spin rotation, following the procedure in ref. 7. From this picture, we derive the effective nuclear precession frequencies as given above. To provide the result for using $m_{\mathrm{s}} = \pm 1$ as the NV electron qubit, we consider a generic model of an NV qubit coupled to a single nuclear spin as $H = |\uparrow\rangle\langle\uparrow| \otimes H_{\uparrow} + |\downarrow\rangle\langle\downarrow| \otimes H_{\downarrow}$, with the conditional nuclear Hamiltonian

$$H_{\uparrow(\downarrow)} = \omega_{\uparrow(\downarrow)}\left(\cos\vartheta_{\uparrow(\downarrow)} I_{\mathrm{z}} + \sin\vartheta_{\uparrow(\downarrow)} I_{\mathrm{x}}\right), \tag{17}$$

where $\omega_{\uparrow(\downarrow)}$ denotes the nuclear precession frequency if the electron spin is at the state $|\uparrow(\downarrow)\rangle$ and $\vartheta_{\uparrow(\downarrow)}$ the corresponding angle of spin alignment. Under the control of an N-pulse CP sequence with the pulse interval τ_{CP}, the conditional evolutions $U_{\uparrow(\downarrow)} = V_{\uparrow(\downarrow)}^{N/2}$ with $V_{\uparrow(\downarrow)} = \exp(-iH_{\uparrow(\downarrow)}\tau_{\mathrm{CP}}/2)\exp(-iH_{\downarrow(\uparrow)}\tau_{\mathrm{CP}})\exp(-iH_{\uparrow(\downarrow)}\tau_{\mathrm{CP}}/2)$. Similar to ref. 7, one can write the unitary evolution as the overall evolution $V_{\uparrow(\downarrow)} = \exp(-i\varphi\mathbf{I}\cdot\hat{\mathbf{n}}_{\uparrow(\downarrow)})$ and the coherence is $L = 1 - (1 - \hat{\mathbf{n}}_{\uparrow}\cdot\hat{\mathbf{n}}_{\downarrow})\sin^2(N\varphi/2)$. From the above definitions, we obtain

$$\cos\varphi = \cos\alpha_{\uparrow}\cos\alpha_{\downarrow} - \cos(\vartheta_{\uparrow} - \vartheta_{\downarrow})\sin\alpha_{\uparrow}\sin\alpha_{\downarrow}, \tag{18}$$

$$1 - \hat{\mathbf{n}}_{\uparrow}\cdot\hat{\mathbf{n}}_{\downarrow} = \frac{4\sin^2(\alpha_{\uparrow}/2)\sin^2(\alpha_{\downarrow}/2)\sin^2(\vartheta_{\uparrow} - \vartheta_{\downarrow})}{1 + \cos\varphi}, \tag{19}$$

with $\alpha_{\uparrow(\downarrow)} = \omega_{\uparrow(\downarrow)}\tau_{\mathrm{CP}}/2$. When $1 - \hat{\mathbf{n}}_{\uparrow}\cdot\hat{\mathbf{n}}_{\downarrow} = 2$ the coherence is maximally modulated by the evolution angle φ of the nuclear spin, giving the resonant condition

$$\cos(\vartheta_{\uparrow} - \vartheta_{\downarrow})\tan(\alpha_{\uparrow}/2)\tan(\alpha_{\downarrow}/2) = 1. \tag{20}$$

We consider the resonance at strong magnetic field that $\gamma_j B_{\mathrm{z}} \gg A_j^{\perp}$, which gives the approximation $\cos(\vartheta_{\uparrow} - \vartheta_{\downarrow}) \approx 1$. Therefore the resonant condition is simplified to $\tan(\alpha_{\uparrow}/2) \approx 1/\tan(\alpha_{\downarrow}/2)$, which has the solutions $\bar{\omega} \equiv (\omega_{\uparrow} + \omega_{\downarrow})/2 \approx (2k+1)\pi/\tau_{\mathrm{CP}}$ with k being integer numbers. Here $(2k+1)\pi/\tau_{\mathrm{CP}}$ are the characteristic frequencies of the NV electron qubit under the control of CP sequence, and $\bar{\omega}$ is interpreted as the effective precession frequency of the nuclear spin. For the case of using the NV qubit states $m_{\mathrm{s}} = 1$ and $m_{\mathrm{s}} = 0$, the effective precession frequency $\bar{\omega} = \gamma_j B_{\mathrm{z}} - \frac{1}{2}A_j^{\parallel}$. For the case of the NV qubit states $m_{\mathrm{s}} = \pm 1$, $\bar{\omega} = \gamma_j B_{\mathrm{z}}$, which is not shifted by the hyperfine field.

c. Shortcomings of spin addressing by dynamical decoupling

The addressing by DD has a number of shortcomings that we are going to discuss in the following.

First, the interaction Hamiltonians achieved by DD do not commute with the nuclear precession. As a consequence, electron-nuclear interactions broaden the nuclear precession frequencies for addressing [see Supplementary Figs. 1 (c) and (d)]. We need the condition in Eq. (16) for individual spin addressing. Reducing the effective interaction strengths between NV electron and nuclear spins improves a lot the spectral resonance by using higher harmonics $k_{\mathrm{DD}} > 1$, by

alternating the phase of Rabi driving[10], or by using composite π pulses[2,4,18,19]. But the reduced coupling also makes nuclear spins that are not strongly coupled hard to detect and control [see Supplementary Figs. 1 (a) and (b)].

Second, because resonances can occur at different harmonics frequencies $k\omega_{\rm DD}$, resonance lines from different harmonic branches can have overlaps and make critical ambiguities in detection and addressing[2,7,20]. The spurious resonances caused by realistic pulse width further complicate the situation, even making false identification of different nuclear species (e.g., ^{13}C and ^{1}H)[20].

Third, the achievable rate $\omega_{\rm DD}$ of DD sets an upper limit on the external magnetic field for spin addressing. A strong magnetic field is the requirement in detecting the chemical shift of nuclear spins[21] and in decoupling of the nuclear dipole-dipole interactions by RF control[3–5]. In addition, the NV electron coherence can be protected easier under strong magnetic fields[22]. However, nuclear spin precession frequencies ω_j at strong magnetic fields can be significantly larger than the achievable rate $\omega_{\rm DD}$ of DD control. For example, the pulse number 35684 required in Supplementary Fig. 1 (d) could be too many in experiments. Using resonance branches with large $k_{\rm DD}$ can reduce the required control rate $\omega_{\rm DD}$, but it also reduces electron-nuclear coupling and narrows spectral bandwidths. As shown in Supplementary Fig. 1 (b) the coupling is too weak to detect the nuclear spin with a weak $A_j^{\perp}$ and the bandwidth is about $\sim \omega_{^{13}\rm C}/k_{\rm DD}$ for ^{13}C spins.

The delayed entanglement echo technique does not suffer from the above shortcomings (compare Supplementary Fig. 2 with Supplementary Fig. 1), and provide some additional advantages. First, it does not require both hyperfine components $A_j^{\parallel}$ and $A_j^{\perp}$ to be strong. Second, both the electron-nuclear coupling strengths before and after the delay window are not reduced, giving better sensitivity. In addition, we can use the levels $m_{\rm s} = \pm 1$ to double the interaction strength (changing $\eta = 1/2$ to $\eta = 1$), since the nuclear spins are addressed by the control in the delay window. In contrast, $\eta = 1/2$ is necessary during the whole protocol of spin addressing by standard DD, for the purpose that homonuclear spins feeling different hyperfine fields have different precession frequencies. Third, our technique allows to simultaneously address more than one nuclear spin by applying RF driving fields at the frequencies of those spins during a delay window.

Supplementary Note 3: Storage of electron states to a memory qubit

Here we present more details on storing the electron qubit states to a nuclear spin memory. During the swap operations, the NV electron qubit is working in the $m_{\rm s} = 0, +1$ manifold. Storage of electron spin state can be realised by SWAP gates. A SWAP gate

$$\text{SWAP} = \sum_{m_{\rm s}, m_{\rm n}=0,1} |m_{\rm s} m_{\rm n}\rangle\langle m_{\rm n} m_{\rm s}| \tag{21}$$

swap the electron qubit states $m_{\rm s}$ and memory qubit states $m_{\rm n}$. In the case that relaxations of the electron and nuclear memory qubit can be neglected during the delay window, we can also use iSWAP gate

$$\text{iSWAP} = \sum_{m_{\rm s}, m_{\rm n}=0,1} e^{i(m_{\rm s}+m_{\rm n})^2\pi/2} |m_{\rm s} m_{\rm n}\rangle\langle m_{\rm n} m_{\rm s}|, \tag{22}$$

which introduces a phase factor i when $m_{\rm s} \neq m_{\rm n}$. Without relaxations of the electron and nuclear memory qubit, the whole system including the environment and the memory qubit has the evolution during the delay window

$$U_{\rm delay} = \sum_{m_{\rm s}, m_{\rm n}=0,1} |m_{\rm s} m_{\rm n}\rangle\langle m_{\rm s} m_{\rm n}| \otimes U_{m_{\rm s}, m_{\rm n}}, \tag{23}$$

where $U_{m_{\rm s}, m_{\rm n}}$ are unitary evolution operators of the environment part. The effect of the iSWAP gates,

$$\text{iSWAP}^{\dagger} U_{\rm delay} \text{iSWAP} = \sum_{m_{\rm s}, m_{\rm n}=0,1} |m_{\rm n} m_{\rm s}\rangle\langle m_{\rm n} m_{\rm s}| \otimes U_{m_{\rm s}, m_{\rm n}}, \tag{24}$$

is the same as using SWAP gates.

We use protected swap gates to suppress decoherence of the NV electron spin and unwanted electron-nuclear interactions during gate implementation. Using nuclear spin addressing by DD[2,4,11,23] or the delayed entanglement echo presented in the main text, we implement the elementary decoherence-protected two qubit gates $u_{{\rm z}\alpha} = \exp\left(i\frac{\pi}{4}\sigma_{\rm z} I_{\alpha}\right)$ with $\alpha = {\rm x, y, z}$ as well as single qubit gates for nuclear spins. Combining the gate $u_{{\rm z}\alpha}$ with electron spin rotations, we achieve the gate $u_{\alpha\alpha} = \exp\left(i\frac{\pi}{4}\sigma_{\alpha} I_{\alpha}\right)$ [e.g., $u_{\rm yy} = \exp\left(i\frac{\pi}{4}\sigma_{\rm x}\right) u_{\rm zy} \exp\left(-i\frac{\pi}{4}\sigma_{\rm x}\right)$]. A swap gate is constructed by $u_{\rm zz} u_{\rm yy} u_{\rm xx}$, where the three gates $u_{\alpha\alpha}$ commute, while $u_{\rm yy} u_{\rm xx}$ gives rise to the iSWAP gate.

a. Storage to the intrinsic nitrogen spin

Here we describe the details of implementation of SWAP gates between the electron qubit and the intrinsic nitrogen spin qubit. For simplicity, we consider ^{14}N, which has 99.636% natural abundance and a spin $I = 1$. The Hamiltonian for the NV electron and the intrinsic nitrogen spins is

$$H_{\rm NV} = DS_{\rm z}^2 - \gamma_{\rm e}B_{\rm z}S_{\rm z} + PI_{\rm z}^2 - \gamma_{\rm N}B_{\rm z}I_{\rm z} + A^{\parallel}S_{\rm z}I_{\rm z} + A^{\perp}(S_{\rm x}I_{\rm x} + S_{\rm y}I_{\rm y}), \tag{25}$$

where $\gamma_{\rm N} = 2\pi \times 0.308$ kHz G^{-1}. We adopt the parameters for ^{14}N in NV centres $A^{\perp} = -2\pi \times 2.62$ MHz, $A^{\parallel} = -2\pi \times 2.162$ MHz, and $P = -2\pi \times 4.945$ MHz[24]. The flip-flop between electron and nuclear spins are suppressed by the large energy mismatch. We have

$$H_{\rm NV} \approx DS_{\rm z}^2 - \gamma_{\rm e}B_{\rm z}S_{\rm z} + PI_{\rm z}^2 - \gamma_{\rm N}B_{\rm z}I_{\rm z} + A^{\parallel}S_{\rm z}I_{\rm z} + \sum_{m_{\rm s}=0,\pm1} |m_{\rm s}\rangle\langle m_{\rm s}|h_{m_{\rm s}}, \tag{26}$$

where the nitrogen operators

$$h_{+1} = \frac{(A^{\perp})^2}{D - \gamma_{\rm e}B_{\rm z}}(|0_{\rm N}\rangle\langle 0_{\rm N}| + |-1_{\rm N}\rangle\langle -1_{\rm N}|), \tag{27}$$

$$h_0 = \frac{(A^{\perp})^2}{-D + \gamma_{\rm e}B_{\rm z}}|+1_{\rm N}\rangle\langle +1_{\rm N}| + \left[\frac{(A^{\perp})^2}{-D + \gamma_{\rm e}B_{\rm z}} - \frac{(A^{\perp})^2}{D + \gamma_{\rm e}B_{\rm z}}\right]|0_{\rm N}\rangle\langle 0_{\rm N}| - \frac{(A^{\perp})^2}{D + \gamma_{\rm e}B_{\rm z}}|-1_{\rm N}\rangle\langle -1_{\rm N}|, \tag{28}$$

$$h_{-1} = \frac{(A^{\perp})^2}{D + \gamma_{\rm e}B_{\rm z}}(|0_{\rm N}\rangle\langle 0_{\rm N}| + |+1_{\rm N}\rangle\langle +1_{\rm N}|), \tag{29}$$

describe the energy shifts caused by virtual spin flip-flop processes.

We use the electron-nitrogen coupling to implement the entangled gate $u_{\rm zz} = \exp(i\frac{\pi}{4}\sigma_{\rm z}\sigma_{\rm N,z})$ in a short duration of 0.23 μs, where $\sigma_{\rm N,z}$ is the Pauli operator of the nitrogen qubit. The iSWAP gate iSWAP $= u_{\rm yy}u_{\rm xx}$, where $u_{\rm xx} = P_{\rm y}u_{\rm zz}P_{\rm y}^{\dagger}$ and $u_{\rm yy} = P_{\rm x}u_{\rm zz}P_{\rm x}^{\dagger}$. Here P_{α} denotes decoherence-protected single-qubit $\pi/2$ gates[23] on both nuclear and electron spins at the α direction. The SWAP gate can be realized by SWAP $= e^{-i\pi/4}u_{\rm zz}$iSWAP.

We simulate the SWAP gate using the protocol and achieve a gate fidelity of $F = 0.987$ (defined by $F = |{\rm Tr}(GU_{\rm g})|/{\rm Tr}(GG^{\dagger})$ with G the evolution of ideal SWAP gate and $U_{\rm g}$ the actual implementation[17]) by taking into account the energy shifts on the electron and nitrogen qubits. The microwave π pulses for the SWAP gate have pulse duration of 12.5 ns, and the field strength for RF $\pi/2$ pulses is 15.53 G. The magnetic field $B_{\rm z} = 0.467$ T and the LG decoupling are the same as those used in Fig. 3b in the main text. In the simulation, we adopt the Hamiltonian Eq. (25) and the electron and nuclear spins feel all the control fields irrespective to their frequencies. Because of the high gate fidelity, we use the nitrogen spin levels $m_{\rm N} = 0, +1$ to store the NV electron qubit by ideal swap gates in producing the figures in the main text that use the nitrogen spin as quantum memory.

After the swap operation, the NV electron spin is polarized to the state $|+1\rangle$ for the delay window. During this storage, we take into account the energy shifts on the nitrogen memory. The energy shifts can also be removed by applying DD (e.g., a Hahn echo) on the nitrogen spin.

b. Storage to carbon-13 memory qubits

We can use AXY sequences[2] to implement $u_{\alpha\alpha}$ gates and swap the NV electron states to a ^{13}C memory. Compared to traditional sequences, AXY exhibits especially good spin addressability, strong robustness against detuning and amplitude errors, and the ability to continuously tune the effective interactions between NV electron and nuclear spins[2]. Using a symmetric version of AXY sequence (see Supplementary Fig. 3 (a)), we have the interaction Hamiltonian $H_{\rm int}^{\rm x} \approx \frac{1}{4}f_{k_{\rm DD}}^{\rm s}A_j^{\perp}\sigma_{\rm z}I_j^{\rm x2}$. Similarly, for anti-symmetric sequences (see Supplementary Fig. 3 (b)), we have $H_{\rm int}^{\rm y} \approx \frac{1}{4}f_{k_{\rm DD}}^{\rm a}A_j^{\perp}\sigma_{\rm z}I_j^{\rm y}$. We tune $f_{k_{\rm DD}}^{\rm s} = f_{k_{\rm DD}}^{\rm a} = f_{k_{\rm DD}}$ and use a time $t_{\rm g} = 2\pi/(f_{k_{\rm DD}}A_j^{\perp})$ to implement the operation

$${\rm iSWAP} = X_{\pi/2}\exp(-iH_{\rm int}^{\rm y}t_{\rm g})X_{\pi/2}^{\dagger}Y_{\pi/2}^{\dagger}\exp(-iH_{\rm int}^{\rm x}t_{\rm g})Y_{\pi/2}, \tag{30}$$

where $X_{\pi/2}$ and $Y_{\pi/2}$ are NV electron $\pi/2$ gates around the directions $\hat{\mathbf{x}}$ and $\hat{\mathbf{y}}$, respectively. The inverse gate iSWAP† can be implemented by the interchanges $X_{\pi/2} \leftrightarrow X_{\pi/2}^{\dagger}$ and $Y_{\pi/2} \leftrightarrow Y_{\pi/2}^{\dagger}$.

Another way to implement the swap gate is by using continuous DD (i.e., using spin-locking field). For the addressed nuclear spin with a distinct precession frequency, we have the effective interaction Hamiltonian $H_{\rm int} \approx \frac{1}{2}\eta A_j^{\perp}(\sigma_{\rm z} I_j^{\rm x} + \sigma_{\rm y} I_j^{\rm y})$ under continuous Rabi driving (see Supplementary Note 2). An iSWAP gate corresponds to the sequence $\exp\left(-i\frac{\pi}{4}\sigma_{\rm y}\right)\exp\left(-iH_{\rm int}t_{\rm g}\right)\exp\left(i\frac{\pi}{4}\sigma_{\rm y}\right)$.

In producing the figures in the main text with a ^{13}C memory, we explicitly implement the swap gate operations by ideal microwave control.

c. *Coherence time of nuclear memory*

A flip of the electron spin changes the magnetic field at the nuclear memory by an amount of the change of the hyperfine field ($A_{\rm m}^{\parallel}$). As a consequence the nuclear memory dephases in a time $\sim 1/A_{\rm m}^{\parallel}$ after an unpredictable flip of the electron spin. It is expected to have one flip of the electron spin in the electron spin relaxation time T_1. Therefore the nuclear memory sufficiently loses its phase coherence after a time $\sim T_1 + 1/A_{\rm m}^{\parallel}$ or $\sim T_1$ if $T_1 \gg 1/A_{\rm m}^{\parallel}$. On the other hand, for a duration $t_{\rm m}$ sufficiently shorter than T_1 we expect no flip of the electron spin and hence the nuclear memory hardly dephases in $t_{\rm m}$, regardless of the strength of the coupling $A_{\rm m}^{\parallel}$.

In Supplementary Fig. 4, we show the coherence of nuclear memories used in the main text, namely, the intrinsic ^{14}N spin and the ^{13}C spin with $A_{\rm m}^{\parallel} = -2\pi\times 31.26$ kHz and $A_{\rm m}^{\perp} = 2\pi\times 29.24$ kHz under a magnetic field of $\gamma_{^{13}{\rm C}}B_{\rm z} = 2\pi\times 5$ MHz. We initialize the NV electron spin to the level $m_{\rm s} = +1$ and the memory qubit in a superposition state that is an eigenstate of the Pauli operator $\sigma_{\rm m,x}$ in $\hat{\mathbf{x}}$ direction. Subsequently we measure the coherence of nuclear memory as the polarization $\sqrt{\langle\sigma_{\rm m,x}\rangle^2 + \langle\sigma_{\rm m,y}\rangle^2}$, without going to the rotating frame of the nuclear spin. The dynamics is solved by Lindblad master equation with the electron spin relaxation rate $1/T_1$. The nuclear spin coherence is compared with the one by the decoherence model of a qubit in a fluctuating environment[25], which uses a model of Markovian noise with the amplitudes given by the hyperfine fields for the corresponding electron spin levels $m_{\rm s}$ that jump with the transition rate $1/T_1$. In Supplementary Fig. 4, the coherence predicted by the classical noise converges to the same curve (solid lines) for both values $A_{\rm m}^{\parallel}$ of the ^{14}N and ^{13}C memories. As shown in Supplementary Fig. 4 the simulations using the Lindblad master equation fit well with the one based on the model of fluctuating environment, confirming the analysis of the nuclear spin decoherence in the above paragraph. Because the nuclear spins have similar coherence times if $A_{\rm m}^{\parallel} > 1/T_1$, we may use a more strongly coupled ^{13}C spin as a quantum memory for fast storage of the NV electron spin state.

Supplementary Note 4: Discussion on electron nuclear double resonance

Some electron nuclear double resonance (ENDOR) techniques utilize RF control to perform spectroscopy on chemical compounds[26]. The stimulated echo [the upper panel of Supplementary Fig. 5 (a)] was recently introduced to detect nuclear spin correlation in a single solid defect centre[27–29]. Because the centre electron spin interacts with all the surrounding nuclear spins in the whole protocol, stimulated echo does not provide advantages on individual spin addressing and control. A modification [which is called Mims ENDOR[30], see Supplementary Fig. 5 (a)] by adding a long RF π pulse on nuclear spins cannot individually address nuclear spins as well, because the electron-nuclear interactions are still not selectively cancelled. In Supplementary Fig. 5 (b) we present the signal of Mims ENDOR when there is only one ^{13}C nuclear spin coupled to the NV electron spin. There are two signal dips of the nuclear spin in spectrum. One at the bare Larmor frequency ($\gamma_{^{13}{\rm C}}B_{\rm z} = 2\pi\times 5$ MHz) of the ^{13}C spin, and another one ($2\pi\times 4980$ kHz) is shifted by the hyperfine field when the electron spin is at the state $m_{\rm s} = +1$. In contrast, in our delayed entanglement echo each single nuclear spin only makes a single signal dip in spectrum, making the nuclear spin signals easier to be identified. In Supplementary Fig. 5 (c) irrespective to the interaction time τ, the population $P = (1 + L)/2$ is always larger than 0.5, which is the case of classical signal[8]. In Supplementary Fig. 5 (d), we show the signal of the same spin when we add one nuclear spin as the spin bath. Although the two nuclear spins have distinct precession frequencies, the signal changes a lot by the perturbation of another nuclear spin, confirming that the Mims ENDOR cannot address and control nuclear spins individually.

The hyperfine-decoupled ENDOR [see Supplementary Fig. 6 (a)] and its improved version (PEANUT ENDOR) [see Supplementary Fig. 6 (b)] proposed in[31] use spin-locking field to lock the electron spin coherence during the application of RF pulse. One may introduce these spin-locking sequences to the field of single defect centres for spin detections. For simplicity, here we calculate the signal when there is only one nuclear spin coupled to the NV centre. Similar to delayed entanglement echo that has no control at the interaction windows, the system evolves for the time τ at each interaction window with the evolution $U_{\rm f} = \exp(-iA^{\parallel}\tau\frac{\sigma_{\rm z}}{2}I_{\rm z})\exp(-i\Omega_{\rm S}\tau\frac{\sigma_{\rm z}}{2})$ in the doubly rotating frame of microwave

and RF control fields. Here $\Omega_{\rm S}$ is the detuning of the spin-locking microwave field. During the spin-locking period, a long RF pulse is applied together with the spin-locking microwave field along the direction $\hat{\mathbf{n}}_{\rm lock}=\sin\theta_{\rm dec}\hat{\mathbf{x}}+\cos\theta_{\rm dec}\hat{\mathbf{z}}$. Here $\cos\theta_{\rm dec}=\Omega_{\rm S}/\Omega$ for the effective locking amplitude Ω and at the present of detuning the angle $\theta_{\rm dec}\neq\pi/2$. The whole evolution of the spin-locking sequences is $U_{\rm off}=U_{\rm f}U_{\rm Lock}U_{\rm f}$ and $U_{\rm on}=U_{\rm f}\exp(-i\pi I_{\rm x})U_{\rm Lock}U_{\rm f}$ for off-resonant and on-resonant RF pulses, respectively. For hyperfine-decoupled ENDOR, $U_{\rm Lock}=\exp(-i\frac{1}{2}\phi_{\rm Lock}\boldsymbol{\sigma}\cdot\hat{\mathbf{n}}_{\rm lock})$, where the rotating angle $\phi_{\rm Lock}=\Omega t_{\rm rf}$ is not prescribed because in typical situations of spin-locking $\phi_{\rm Lock}\gg 1$ and a small control fluctuation can have a significant change of $\phi_{\rm Lock}$ larger than 2π. In these spin-locking sequences, the electron spin is initially prepared in a superposition state by a $\pi/2$ pulse around the $\hat{\mathbf{x}}$ direction, and the electron spin polarization signal along the $\hat{\mathbf{y}}$ direction is averaged over $\phi_{\rm Lock}$ to gives the signal. For on-resonant RF driving, the signal of hyperfine-decoupled ENDOR is[31]

$$E_{\rm rf}=-\sin^2\theta_{\rm dec}\left[\cos^2\left(\frac{A^{\parallel}\tau}{2}\right)\sin^2(\Omega_{\rm S}\tau)-\sin^2\left(\frac{A^{\parallel}\tau}{2}\right)\cos^2(\Omega_{\rm S}\tau)\right]. \tag{31}$$

While for off-resonant RF driving, the signal[31]

$$E_{0}=-\sin^2\theta_{\rm dec}\left[\cos^2\left(\frac{A^{\parallel}\tau}{2}\right)\sin^2(\Omega_{\rm S}\tau)+\sin^2\left(\frac{A^{\parallel}\tau}{2}\right)\cos^2(\Omega_{\rm S}\tau)\right], \tag{32}$$

shows that nuclear spins are not addressed selectively by hyperfine-decoupled ENDOR because nuclear spins under off-resonant RF driving still perturb the electron spin dynamics. In contrast, in our delayed entanglement echo nuclear spins under off-resonant RF driving do not perturb the NV dynamics and do not module the detection signal, which are fundamental requirements for individual addressing of nuclear spins. For PEANUT ENDOR, $U_{\rm Lock}=I$ is an identity evolution when there is no detuning error $\Omega_{\rm S}=0$, regardless of the frequencies of nuclear spins. Therefore, as the case of hyperfine-decoupled ENDOR, one cannot use PEANUT ENDOR to individually addressed a number of nuclear spins, because in these ENDOR sequences the NV electron couples to all nuclear spins in an unselective manner.

In summary, these ENDOR techniques are not suitable for single spin addressing and control. Other shortcomings of hyperfine-decoupled ENDOR in the application to single-defect centres are the high-power consumption of microwave as well as electron dephasing and relaxation due to fluctuations of the spin-locking field.

Supplementary Note 5: Details of simulations

a. General remarks.

The ^{13}C spins of the diamond samples are randomly distributed around the NV centre. In simulations for NV dynamics, we randomly distribute ^{13}C spins around the NV centre and select samples that do not contain ^{13}C nuclei within a distance of 0.714 nm from the NV centre (corresponding to 274 atomic sites), so that the hyperfine interactions between the ^{13}C nuclei and NV electron spin are simply described by the dipolar coupling. The probability of getting this kind of samples is $\sim 5\%$ for natural abundance of 1.1% and is higher for lower abundances. Because of low spin concentration, simulations are accurate enough by grouping nuclear spins into interacting clusters and neglecting the intercluster interactions[32]. Nuclear spins are initially in thermal mixed states if they are not polarized.

The microwave and RF control fields takes the form $(B_{\rm c}^{\rm x}\hat{\mathbf{x}}+B_{\rm c}^{\rm z}\hat{\mathbf{z}})\cos(\omega_{\rm c}t+\phi_{\rm c})$ in the simulations. Non-vanishing misalignments $B_{\rm c}^{\rm z}/B_{\rm c}^{\rm x}>0.125$ are introduced to mimic general experimental situations. The Rabi frequency is determined by the values of $B_{\rm c}^{\rm x}$. Because of the control fields, the total Hamiltonians is time-dependent. To simulate the control fields, we sample the control fields in a time step of the minimum values of $0.01\times 2\pi/\omega_{\rm c}$. In the simulations, we choose the intervals of successive π pulses as multiples of the periods of RF driving field $2\pi/\omega_{\rm rf}$.

We adopt the coordinate system $\hat{\mathbf{z}}=[111]/\sqrt{3}$ along the symmetry axis of NV centre and the orthogonal unit vectors $\hat{\mathbf{x}}=[1\bar{1}0]/\sqrt{2}$ and $\hat{\mathbf{y}}=[11\bar{2}]/\sqrt{6}$ to record the positions of ^{13}C spins $\mathbf{r}_j=[\mathbf{r}_j\cdot\hat{\mathbf{x}},\mathbf{r}_j\cdot\hat{\mathbf{y}},\mathbf{r}_j\cdot\hat{\mathbf{z}}]$, which are measured relative to the location of the NV electron spin at the origin $[0,0,0]$.

b. Nuclear spin bath.

The sample used for Fig. 2 in the main text has a natural abundance of 1.1% and contains the host nitrogen and 736 ^{13}C nuclei, by neglecting weakly coupled ^{13}C nuclei at distances larger than 4.5 nm. To simulate the dynamics using the method in ref. 32, we group the nuclei into interacting clusters by neglecting intercluster interactions $\leq 2\pi\times 70$ Hz,

giving spin clusters with up to 7 spins. For this sample, a spin echo π pulse extends the electron coherence times from $T_2^* \approx 4$ μs to ~ 1 ms under magnetic fields much larger than the hyperfine fields at the nuclei (see Supplementary Fig. 7), consistent with theories[22] and experiments[33]. Supplementary Fig. 7 also shows that the NV coherence time can be much longer if the nuclear-nuclear interactions are suppressed. To make the simulation feasible, we assume instantaneous ideal π pulses on the NV electron spin, and we apply secular approximation on the NV electron spin by using that the NV electron spin can not be flipped by nuclear spins in the relevant time scales.

c. *High-fidelity quantum gates.*

In Fig. 3 of the main text, the gate fidelity is calculated by $F = |\mathrm{Tr}(UQ^\dagger)|/|\mathrm{Tr}(QQ^\dagger)|$[17] for the target unitary quantum gate Q and the evolution U on the ^{13}C spins G1 ($\mathbf{r}_{\mathrm{G1}} = [0.80325, 0.80325, 0.44625]$ nm), G2 ($\mathbf{r}_{\mathrm{G2}} = [-0.62475, -0.80325, -0.80325]$ nm), and the nitrogen spin in the rotating frame. With the nuclear qubit G1 addressed with $\theta_{\mathrm{rf}} = \pi$, the target gate

$$Q = [U_{\mathrm{ent}}(\phi_{\mathrm{g}}) e^{-i\pi I^{x}_{\mathrm{G1}}}] \otimes I_{\mathrm{G2}} \otimes I_{^{14}\mathrm{N}}, \tag{33}$$

is an entanglement quantum gate, where the identities I_{G2} and $I_{^{14}\mathrm{N}}$ represent no-operation (NULL) gates on the unaddressed spins G1 and the nitrogen, respectively. For interaction time $\tau = 0$ and a rotation angle θ_{rf} the gate becomes single-qubit gates

$$Q = I_{\mathrm{e}} \otimes e^{-i\theta_{\mathrm{rf}} I^{x}_{\mathrm{G1}}} \otimes I_{\mathrm{G2}} \otimes I_{^{14}\mathrm{N}}, \tag{34}$$

with the identity gate on the electron qubit. The DD sequences applied at the delay window are equally-spaced AXY-8 sequences with a total number of 80 rectangular π pulses. The echo π pulse after the delay and before the second interaction widows is implemented by a composite Knill pulse (consisting of 5 elementary π pulses)[34]. In simulations for quantum gates, we apply a composite Knill pulse at the end of the protocol. All the π pulses have a pulse length of 50 ns and contain amplitude errors and hence rotation errors. In simulations for population signal, we apply rectangular $\pi/2$ pulses (pulse duration 25 ns, no amplitude error) before and after the delayed entanglement echo for state preparation and readout. In the simulation, we do not polarize the nuclear spins. Therefore the ^{14}N spin initially in a thermal mixed state causes a detuning error of $\sim 2\pi\times 2.2$ MHz on all the microwave pulses through the hyperfine coupling.

d. *Electron spin relaxation.*

In simulations for the results of Fig. 4a in the main text, the electron spin relaxation is solved by Lindblad master equations, using an electron spin relaxation rate $1/T_1$. The effects of electron spin relaxation on the nuclear spin dephasing are discussed in Supplementary Note 3. In Fig. 4a of the main text, the signals come from the addressing to an isolated ^{13}C nucleus located at $\mathbf{r}_j = [0.0, -1.9635, -0.8925]$ nm with the weak hyperfine coupling $A_j^{\parallel} = 2\pi \times 1.49$ kHz and $A_j^{\perp} = 2\pi \times 2.93$ kHz. The ^{13}C memory qubit located at $[-0.714, 0.0, 0.357]$ nm has $A_{\mathrm{m}}^{\parallel} = -2\pi \times 31.26$ kHz and $A_{\mathrm{m}}^{\perp} = 2\pi \times 29.24$ kHz. AXY sequences with instantaneous π pulses are used to simulate the iSWAP gate on the ^{13}C qubit with a gate time $2t_{\mathrm{g}} \approx 318$ μs, using a total number of ~ 152 composite π pulses (explicitly, 760 elementary π pulses since one composite pulse in AXY sequences has 5 elementary π pulses). See Supplementary Note 3 for the details of the swap operations on the ^{14}N or the ^{13}C nuclear spin memories.

e. *Nuclear spin pairs.*

In Fig. 4b of the main text, the two ^{13}C nuclei in a C-C bond are located at $\mathbf{r}_j = [-1.2495, 0.714, -0.1785]$ nm and $\mathbf{r}_k = [-1.33875, 0.80325, -0.26775]$ nm, which imply a dipolar coupling of $d_{j,k} = 2\pi \times 1.37$ kHz. The hyperfine components $A_j^{\parallel} = -2\pi \times 4.94$ kHz and $A_j^{\perp} = 2\pi \times 5.33$ kHz for spin j, while for another spin k $A_k^{\parallel} = -2\pi \times 3.72$ kHz and $A_k^{\perp} = 2\pi \times 4.2$ kHz. In Fig. 5a,c of the main text, the two ^{13}C nuclei are located at $\mathbf{r}_j = [0, -0.8925, -0.5355]$ nm and $\mathbf{r}_k = [-0.08925, -0.80325, -0.62475]$. In Fig. 5b,d of the main text, the two ^{13}C nuclei are located at $\mathbf{r}_j = [-1.071, -0.357, 1.071]$ nm and $\mathbf{r}_k = [-1.16025, -0.26775, 0.98175]$, and we adopt the approach that uses a ^{14}N memory in the simulations.

f. Interaction windows protected by DD.

In Fig. 4c of the main text, the two separated spins located at $\mathbf{r}_j = [0.0, -1.9635, -0.8925]$ nm (i.e., the target spin in Fig. 4a of the main text) and $\mathbf{r}_k = [0.0, 1.2495, 1.9635]$ nm have similar hyperfine components. The hyperfine values for the second weakly coupled spin are $A_k^{\|} = 2\pi \times 1.43$ kHz and $A_k^{\perp} = 2\pi \times 2.28$ kHz. We protect the interaction window with $\tau = 0.5$ ms, by using CP sequences with 1000 microwave π pulses (corresponding to 200 composite π pulses for AXY sequences). In the delay window, the two CP π pulses on the ^{13}C spins have the pulse duration $250 \times 2\pi/\omega_{\rm rf}$, corresponding to a Rabi frequency of $\approx 2\pi \times 10$ kHz for $\omega_{\rm rf} \approx 2\pi{\times}5$ MHz in the simulations.

g. Decoupling by optical illumination.

In the simulation with optical illumination used in Fig. 4d of the main text, we adopt the Lindblad model of the experimental paper[35]. This model provides results in good agreement with experimental data[35], by incorporating 11 levels for the NV electronic states to describe the optical transitions, electronic decay, spin relaxation, as well as the ionization and deionization of NV centre. To use the model for our system, we replace the ^{13}C memory spin in ref. 35 by our ^{13}C memory spin and add a proton spin for detection. The memory ^{13}C spin is similar to the one used in ref. 35, with the location $\mathbf{r}_{\rm m} = [-0.108, -0.295, -1.74]$ nm and hyperfine components $A_{\rm m}^{\|} = -2\pi \times 1.69$ kHz and $A_{\rm m}^{\perp} = 2\pi{\times}5.4$ kHz. The proton spin for detection is located 4 nm away from the NV centre, with $\mathbf{r}_j = [2.31, 2.31, 2.31]$ nm (hence $A_j^{\perp} = 0$ and the spin is hard to detect by standard DD). We adopt the parameters in ref. 35. For the tunable parameter of the laser intensity, we use a value of $64I_{\rm sat}$ (in terms of saturation intensity $I_{\rm sat}$), which gives the deionization rate $\gamma_1 = 64/(70{\rm ns})$ and ionization rate $\gamma_2 = 2\gamma_1$ used for simulations[35]. Note that ref. 35 also confirms that the presence of RF driving does not interfere in the optical illumination process, because of their very different operating frequencies.

In performing the iSWAP gates for the protocol we use continuous DD[36] on the NV electron qubit with the Rabi frequency on resonant to the precession frequency of the ^{13}C memory. To implement a complete SWAP gate, we use delayed entanglement echo on the ^{13}C memory after the iSWAP operation. The delay window for the ^{13}C memory uses a 20-pulse CP sequence with duration ≈ 100 μs for a protected RF π gate. The total SWAP gate time for the simulation is ≈ 584 μs, which can be reduced by a factor of two if we use the electron levels $m_{\rm s} = \pm 1$. The gate can be further protected by storing the electron state to the nitrogen spin when applying the delay window for the ^{13}C memory. Using a ^{13}C memory more strongly coupled to the NV electron can also significantly reduce the required SWAP gate times.

The procedure to detect the chemical shifts of proton spins with optical illumination is the following. We first pump the NV electron spin by optical field to initialize the NV electron spin to the state $|0\rangle$ with a fidelity 82% (the fidelity is obtained for the parameters in ref. 35 and it is higher for better samples), which is followed by using a swap operation to polarize the ^{13}C memory spin. Then we use optical pumping again and a microwave pulse to initialize the NV electron to a superposition state $(|0\rangle + |+1\rangle)/\sqrt{2}$. After the initialization of NV electron spin and memory qubit, we let the whole system freely evolve for a time of τ to generate electron-nuclear entanglement (which can be protected by DD as shown in the main text). Then we store the NV electron spin to the memory spin by a swap gate. Subsequently we use optical illumination to decouple electron-nuclear coupling for applying a RF pulse with the length $t_{\rm rf} \approx 80$ ms. The carry frequency of RF driving is set to the target proton spin. After optical illumination, we wait for 2 μs to relax the NV electron spin back to $|0\rangle$ state. Subsequently, we use a swap gate to retrieve the quantum state of NV electron spin and to re-popularize the ancillary ^{13}C spin. Finally, we apply a microwave π pulse on the electron spin and wait for another interaction window of time τ before readout of the electron spin state. We can increase the interaction to target spins by using the NV levels $m_{\rm s} = \pm 1$, as described in the main text.

Supplementary References

[1] Doherty, M. W. *et al.* The nitrogen-vacancy colour centre in diamond. *Phys. Rep.* **528,** 1-45 (2013).

[2] Casanova, J., Wang, Z.-Y., Haase, J. F. & Plenio, M. B. Robust dynamical decoupling sequences for individual-nuclear-spin addressing. *Phys. Rev. A* **92,** 042304 (2015).

[3] Lee, M. & Goldburg, W. I. Nuclear-magnetic-resonance line narrowing by a rotating rf field. *Phys. Rev.* **140**, A1261-A1271 (1965).

[4] Wang, Z.-Y., Haase, J. F., Casanova, J. & Plenio, M. B. Positioning nuclear spins in interacting clusters for quantum technologies and bioimaging. *Phys. Rev. B* **93,** 174104 (2016).

[5] Cai, J., Retzker, A., Jelezko, F. & Plenio, M. B. A large-scale quantum simulator on a diamond surface at room temperature. *Nature Phys.* **9,** 168-173 (2013).

[6] Kolkowitz, S., Unterreithmeier, Q. P., Bennett, S. D. & Lukin, M. D. Sensing distant nuclear spins with a single electron spin. *Phys. Rev. Lett.* **109,** 137601 (2012).

[7] Taminiau, T. H. *et al.* Detection and control of individual nuclear spins using a weakly coupled electron spin. *Phys. Rev. Lett.* **109,** 137602 (2012).

[8] Zhao, N. *et al.* Sensing single remote nuclear spins. *Nature Nanotech.* **7,** 657-662 (2012).

[9] London, P. *et al.* Detecting and polarizing nuclear spins with double resonance on a single electron spin. *Phys. Rev. Lett.* **111,** 067601 (2013).

[10] Mkhitaryan, V. V., Jelezko, F. & Dobrovitski, V. V. Highly selective detection of individual nuclear spins with rotary echo on an electron spin probe. *Sci. Rep.* **5,** 15402 (2015).

[11] Taminiau, T. H., Cramer, J., van der Sar, T., Dobrovitski, V. V. & Hanson, R. Universal control and error correction in multi-qubit spin registers in diamond. *Nature Nanotech.* **9**, 171-176 (2014).

[12] Liu, G.-Q., Po, H. C., Du, J., Liu, R.-B. & Pan, X.-Y. Noise-resilient quantum evolution steered by dynamical decoupling. *Nature Commun.* **4**, 2254 (2013).

[13] Carr, H. Y. & Purcell, E. M. Effects of diffusion on free precession in nuclear magnetic resonance experiments. *Phys. Rev.* **94,** 630-638 (1954).

[14] Meiboom, S. & Gill, D. Modified spin-echo method for measuring nuclear relaxation times. *Rev. Sci. Instrum.* **29,** 688-691 (1958).

[15] Maudsley, A. A. Modified Carr-Purcell-Meiboom-Gill sequence for NMR Fourier imaging applications. *J. Magn. Reson.* **69,** 488-491 (1986).

[16] Gullion, T., Baker, D. B. & Conradi, M. S. New, compensated Carr-Purcell sequences. *J. Magn. Reson.* **89,** 479-484 (1990).

[17] Casanova, J., Wang, Z.-Y. & Plenio, M. B. Noise-resilient quantum computing with a nitrogen-vacancy center and nuclear spins. *Phys. Rev. Lett.* **117,** 130502 (2016).

[18] Zhao, N., Wrachtrup, J., & Liu, R.-B. Dynamical decoupling design for identifying weakly coupled nuclear spins in a bath. *Phys. Rev. A* **90,** 032319 (2014).

[19] Ma, W. *et al.* Resolving remote nuclear spins in a noisy bath by dynamical decoupling design. *Phys. Rev. A* **92,** 033418 (2015).

[20] Loretz, M. *et al.* Spurious harmonic response of multipulse quantum sensing sequences. *Phys. Rev. X* **5,** 021009 (2015).

[21] Mehring, M. *Principle of High Resolution NMR in Solids* (Springer, New York, 1983).

[22] Zhao, N., Ho, S.-W. & Liu, R.-B. Decoherence and dynamical decoupling control of nitrogen vacancy center electron spins in nuclear spin baths. *Phys. Rev. B* **85,** 115303 (2012).

[23] van der Sar, T. *et al.* Decoherence-protected quantum gates for a hybrid solid-state spin register. *Nature* **484,** 82-86 (2012).

[24] Chen, M., Hirose, M. & Cappellaro, P. Measurement of transverse hyperfine interaction by forbidden transitions. *Phys. Rev. B* **92,** 020101(R) (2015).

[25] Kitajima, S., Ban, M. & Shibata, F. Theory of decoherence control in a fluctuating environment. *J. Phys. B: At. Mol. Opt. Phys.* **43,** 135504 (2010).

[26] Schweiger, A. & Jeschke, G. *Principles of Pulse Electron Paramagnetic Resonance* (Oxford, New York, 2001).

[27] Laraoui, A. *et al.* High-resolution correlation spectroscopy of ^{13}C spins near a nitrogen-vacancy centre in diamond. *Nature Commun.* **4,** 1651 (2013).

[28] Staudacher, T. *et al.* Probing molecular dynamics at the nanoscale via an individual paramagnetic centre. *Nature Commun.* **6,** 8527 (2015).

[29] Kong, X. *et al.* Towards chemical structure resolution with nanoscale nuclear magnetic resonance spectroscopy. *Phys. Rev. Applied* **4,** 024004 (2015).

[30] Mins W. B. Pulsed endor Experiments. *Proc. Royal Soc. Lond. Math. Phys. Engineer. Sci.* **283,** 452-457 (1965).

[31] Jeschke G. & Schweiger, A. Hyperfine decoupling in electron spin resonance. *J. Chem. Phys.* **106,** 9979-9991 (1997).

[32] Maze, J. R., Taylor, J. M. & Lukin, M. D. Electron spin decoherence of single nitrogen-vacancy defects in diamond. *Phys. Rev. B* **78,** 094303 (2008)

[33] Cramer, J. *et al.* Repeated quantum error correction on a continuously encoded qubit by real-time feedback. *Nature Commun.* **7,** 11526 (2016).

[34] Ryan, C. A., Hodges, J. S. & Cory, D. G. Robust decoupling techniques to extend quantum coherence in diamond. *Phys. Rev. Lett.* **105,** 200402 (2010).

[35] Maurer, P. C. *et al.* Room-temperature quantum bit memory exceeding one second. *Science* **336,** 1283-1286 (2012).

[36] London, P. *et al.* Detecting and polarizing nuclear spins with nuclear double resonance on a single electron spin. *Phys. Rev.*

Lett. **111,** 067601 (2013).
